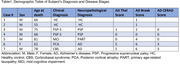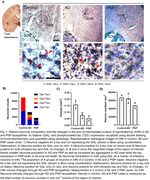# The VLPO analog in the human hypothalamus shows tau‐driven extreme loss of sleep‐regulating neurons in PSP and Alzheimer’s disease: unveiling the basis of NREM sleep dysfunction in tauopathies

**DOI:** 10.1002/alz.084555

**Published:** 2025-01-03

**Authors:** Shima Rastegar‐Pouyani, Caroline Lew, Abhijit Satpati, Renata Elaine Paraizo Leite, Claudia Kimie Suemoto, Salvatore Spina, William W. Seeley, Christine M Walsh, Thomas C. Neylan, Lea T. Grinberg

**Affiliations:** ^1^ UCSF, San Francisco, CA USA; ^2^ Memory and Aging Center, Weill Institute for Neurosciences, University of California San Francisco, San Francisco, CA USA; ^3^ Memory and Aging Center, UCSF Weill Institute for Neurosciences, University of California San Francisco, San Francisco, CA USA; ^4^ University of São Paulo Medical School, São Paulo Brazil; ^5^ University of São Paulo Medical School, São Paulo, São Paulo Brazil; ^6^ University of California, San Francisco, San Francisco, CA USA; ^7^ Weill Institute for Neurosciences and Memory and Aging Center, Department of Neurology, University of California, San Francisco, CA USA; ^8^ Weill Institute for Neurosciences, University of California San Francisco, San Francisco, CA USA

## Abstract

**Background:**

Sleep dysfunction is commonly seen in Alzheimer’s disease (AD) and Progressive Supranuclear Palsy (PSP), potentially worsening these conditions. Investigating early neuropathological changes in human sleep‐promoting neurons, which often precede cognitive decline, is crucial for understanding the basis for sleep dysfunction as possible treatments yet remain underexplored. We used postmortem brains of AD and PSP patients to quantify neuronal numbers and tau burden in the intermediate nucleus of the hypothalamus (IntN), VLPO analog, known for its role in sleep maintenance.

**Method:**

Postmortem human brain tissues from three groups were used: healthy controls, progressive AD stages, and PSP. Formalin‐fixed, celloidin‐embedded hypothalamic tissue was cut at 30‐micron. Slides were immunostained for galanin (GAL), an IntN neuronal marker, phosphorylated‐tau (p‐tau) antibodies, and counterstained with Gallocyanin. The total number of GAL‐expressing neurons and/or p‐tau deposition were estimated using the stereology.

**Results:**

In a cohort of 7 cases (Table 1), we found a significant decrease in neuronal population and volume in the IntN in both AD and PSP patients, with a more pronounced decline in PSP. However, the neuronal density was not changed in any of the AD or PSP conditions, reflecting a simultaneous neuronal and volume loss. GAL‐positive neurons were more resilient in AD, showing better preservation than in PSP. In both AD and PSP, p‐tau‐positive neurons increased compared to the controls. Notably, in AD, tau aggregates were present in both GAL‐positive and negative neurons, while in PSP, GAL neuronal population decreased more than the others, and tau aggregates were only observed in GaL‐negative neurons (Fig. 1).

**Conclusion:**

In both AD and PSP, our research reveals substantial neuropathological changes in the IntN, with PSP exhibiting more pronounced alterations. These changes align with observed sleep dysfunction in both conditions. PSP features severe insomnia, although wake‐promoting neurons remain relatively intact, thus a significant reduction in neurons controlling NREM‐sleep could account for its clinical sleep disturbances. Conversely, AD displays a decline in wake‐promoting neurons and a gradual, moderate loss of NREM neurons, both leading to excessive daytime sleepiness and sleep fragmentation, yet overall preserved total sleep time. These findings deepen our understanding of NREM sleep dysfunction in tauopathies, offering insights for targeted interventions.